# Understanding social dynamics and patient experience in out of hospital care: validation of a co-responsibility questionnaire (CoReCare)

**DOI:** 10.1038/s41598-024-54147-0

**Published:** 2024-02-28

**Authors:** Melanie Knufinke-Meyfroyt, Carlo Lancia, Yentl Lodewijks, Simon Nienhuijs, Eva Deckers

**Affiliations:** 1 Philips Experience Design, Eindhoven, The Netherlands; 2 Department of Surgery, Catharina Hospital, Eindhoven, The Netherlands; 3 Program Management Digitization, Catharina Hospital, Eindhoven, The Netherlands

**Keywords:** Psychology, Health care

## Abstract

Patient experiences are commonly assessed through patient reported experience measures (PREMs). Ambulatory care models extend traditional care into the patients’ home, meaning that a triangle of health care professionals, patients, and their families need to be considered when assessing the remote care experience. These intertwined responsibilities are described by co-responsibility. Currently, PREMs don’t reflect how elements to remote care impact this remote care experience. Therefore, this study aimed to develop a questionnaire assessing perceived patient-partner co-responsibility as a PREM in remote care. A 30-item questionnaire was assessed among 1000 individuals aged between 18 and 65 years that tried to lose weight with a partner, friend or family member supporting them. Pairwise item correlations, Exploratory Factor Analysis, and Cronbach’s alpha were used for validation. 29-items were identified to reflect co-responsibility across 6 factors: empowerment and support, relational aspects, lack of sympathy, co-participation, accepting help and awareness. Cronbach’s alpha ranged between 0.66 and 0.93, showing good internal consistency. We present a validated CoReCare Questionnaire to understand the impact of social dynamics on achieving desired health outcomes in a remote care setting. The CoReCare Questionnaire extends current PREMs when aiming to assess and improve the patient experience of a care episode outside of the hospital.

## Introduction

Patient experience of care is fundamental to the quality of healthcare^1^ and has repeatedly been linked to better health outcomes^2,3^. The transition to value-based healthcare (VBC) models^4^, the increase in remote care innovations^5^, and health behavior change programs are altering traditional care pathways. In remote care, additional factors like the involvement of a patient’s family can impact their experience of care. These dynamics need to be understood and adequately reflected in methodologies assessing remote patient experience, health outcomes, and quality of care.

VBC models focus on improving quality of care, patient and staff experience, and reducing cost of care^4^. Responding to this trend, methods have been developed to identify areas of improvements and to measure the effectiveness of patient-centered interventions^2,3,6^. The gold standard of assessing patient experience are Patient Reported Experience Measures (PREMS)^7^. PREMS can be generic, i.e., assessing the experience of care processes and the quality of caretaker interactions^6,7^, or tailored to a specific care context^8^.

Parallel to this surge of patient-centered care is the rise of remote (after) care. Part of the remote care propositions encompass programs on behavioral change in which patients aim to adopt a healthier lifestyle guided by healthcare professionals. For example, in stomach reduction (bariatric/metabolic) surgery, patients follow a multi-disciplinary aftercare program, focusing on a six-meals a day, a protein enriched diet and physical activity to reduce the risk of regaining weight^9,10^.

The introduction of home-based interventions and after care programs is extending the traditional care journey into the home of patients. Therefore, responsibilities are shifting from medical professionals to significant others in the home environment of patients. For example, in hospital-based care settings, patients interact with health care professionals and receive advice tailored to their care needs. Whereas at home, patients are surrounded by partners, family, and friends, who might unconsciously influence their lifestyle choices and health behaviors. Thus, one should consider a triangle between health care professionals, patients, and their family and friends when assessing the experience of remote care^11^. The importance of social dynamics on health behaviors has been acknowledged in interventions and questionnaires on social- and spousal support^12^. Yet, current PREMS might not reflect well enough whether and how elements unique to the remote care settings impact the experience of a care journey.

Devisch introduced ‘co-responsibility’ to describe these intertwined responsibilities between patients, their partners, families, and health care professionals in achieving health goals^13^. The concept has been applied in a field study among bariatric surgery patients and their partners^14^ and has been operationalized and tested in a survey among bariatric patients^15^. However, as stated by the authors, parts of the construct—like ‘desired co-responsibility’—needs refinement to adequately reflect all aspects of co-responsibility and to establish better psychometric parameters.

While co-responsibility resembles components of spousal support, such as relational aspects, empowerment and support, and co-participation, we believe it extents this notion by addressing the ability to accept help and perceived empathy. Most strongly co-responsibility extends spousal support by addressing the bi-directional impact of behavioral patterns among patients and individuals in close proximity. This study aims to understand the extent to which co-responsibility resembles or augments spousal support, since this has not been empirically tested.

This study aimed to develop a refined questionnaire to assess perceived co-responsibility as an important element of PREMs in remote care setting. We aimed (1) to test psychometric properties, and (2) to identify the underlying factor structure of a questionnaire to assess perceived co-responsibility among 1000 individuals attempting to lose weight through adopting healthier lifestyle behaviors. While intending to develop a tool for (bariatric) patients, we chose a sample of healthy individuals targeting weight loss to reduce the burden of early-stage research participation among patients.

## Data and methods

We collected data in December 2021 through an online survey hosted by the market research agency Ipsos MORI, Amsterdam, The Netherlands. A sample of healthy individuals targeting weight loss was chosen to reduce the burden of early-stage research participation among patients. Individuals were eligible for the study if (1) they provided digital informed consent, (2) attempted to lose weight for at least the past 4 weeks, (3) were not pregnant, receiving medical treatment that affects body weight, or had undergone stomach reduction surgery, (4) had a partner, friend, or family member that supported them in their weight loss journey, (5) were aged between 18 and 65 years, and (6) completed all mandatory questions. We gathered responses until a total sample of 1000 respondents was achieved. The final sample comprised 510 women and 490 men, with an average age of 53 years (SD = 10, ranging between 21 and 65 years). The average BMI was 30 (SD = 4) and participants intended to lose 16 kg of weight on average (SD = 12).

The study was approved by the Philips Internal Committee for Biomedical Experiments [ICBE-S-000711] and was performed in accordance with the Declaration of Helsinki. All participants provided informed consent through a build-in click-through consenting procedure and could pause, stop, or terminate participation at any time.

### Investigational questionnaire

To assess co-responsibility, we developed a 30-item questionnaire. In close collaboration with clinical partners in bariatric surgery, the questionnaire was built on insights from a field-study implementing the concept of co-responsibility^14^ and a former co-responsibility questionnaire^15^. The former 19 items questionnaire was validated among 390 metabolic surgery patients and associations between co-responsibility, spousal support, and health outcomes such as weight loss, eating and exercise habits, and self-efficacy were established. Results from Principal Component Analysis (PCA) revealed three components reflecting co-responsibility: ‘‘Satisfaction with partner support’’, ‘‘Love and support’’, and ‘‘Desired co-responsibility’’, with high internal consistency, except for ‘‘Desired co-responsibility’’.

Items of this new questionnaire were designed to yield better internal consistency for a strong and adequate reflection of co-responsibility. Items could be scored on a 5-point Likert scale ranging from ‘1—fully disagree’ to ‘5—fully agree’.

To verify construct validity of co-responsibility, we assessed spousal support using the shortened, 10-item version of the Close Persons Questionnaire (CPO^16^).

### Statistical methods

Five-point Likert scales were converted to integers ranging from 0 to 4. Negatively phrased items (I17, I25, I26, I27, and I30 in Table 2) were further processed by inverting the converted score around the value 2 (0 → 4, 1 → 3, etc.). We assessed pairwise item relationships using Pearson’s $\rho$ correlation coefficient.

We investigated the underlying structure of the co-responsibility sub-questionnaire items through Exploratory Factor Analysis. We determined the number of factors using Kaiser criterion^17^ and Cattell scree test^18^. As factors are expected to be correlated, we selected the oblique rotation which maximizes the explained variance. Factor loadings below 0.4 in absolute value were not included in the process of mapping items onto constructs. We assessed the internal consistency of the elementary constructs identified in this step through Cronbach’s $\alpha$ coefficient^19^.

For each respondent, we summed the scores of the items belonging to a construct to obtain the score of that construct; in particular, the co-responsibility score was obtained by summing the scores of items from I01 to I30, see Table 2. We further explored pairwise relationships among the constructs using binned scatterplots and computing Pearson’s $\rho$ correlations. The vertical dispersion of the binned scatterplot is very small in the region where the factor scores are large. We also calculated Pearson’s rho between the spousal support score (defined as the sum of the answers’ face value of the 10-item CPO questionnaire) and the co-responsibility score (defined as the sum of the answers of the co-responsibility items loading above the 0.4 threshold).

Estimation uncertainty is always represented by a 95% confidence interval. All analyses were performed using Python 3.10.4^20^ with NumpY 1.21.6^21^, SciPy 1.8.1^22^, Statsmodels 0.13.2^23^, Pingouin 0.5.2^24^, Scikit-learn 1.1.1^25^, Matplotlib 3.5.1^26^, and M. seaborn 0.11.2^27^.

## Results

**Figure 1:**
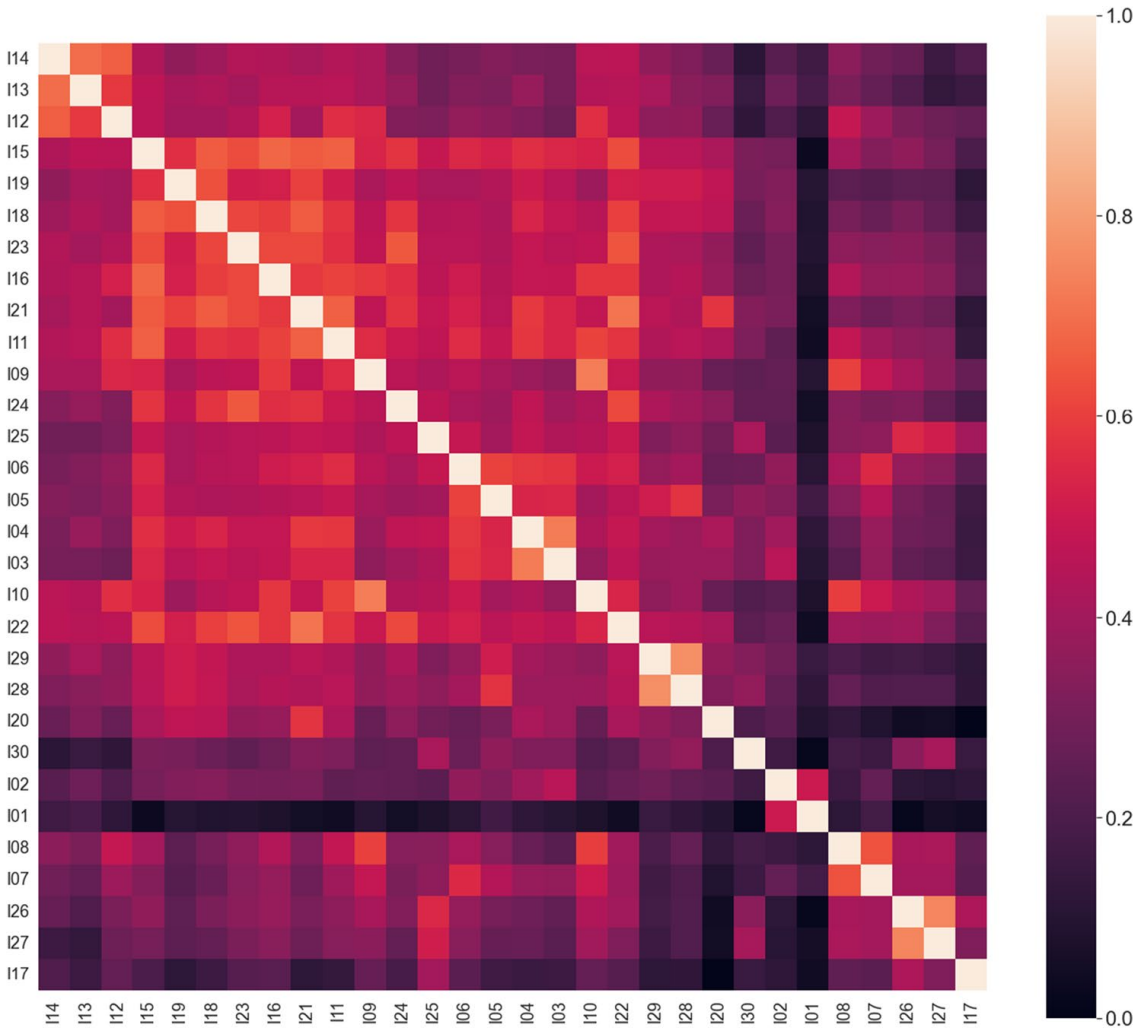
Correlation matrix for the questionnaire items (after inversion of negatively phrased questions). Codes are mapped to items in the first column of Table 2. Since items are sorted into groups of similar correlations, it is already possible to see some of the structures that are identified by factor analysis, e.g. I12, I13, and I14 (Factor 5), or I17, I26, and I27 (contributing to Factor 3).

Correlations between items are shown as a heatmap in Fig. 1. As expected, all correlations are non-negative, validating the inversion procedure.

**Figure 2:**
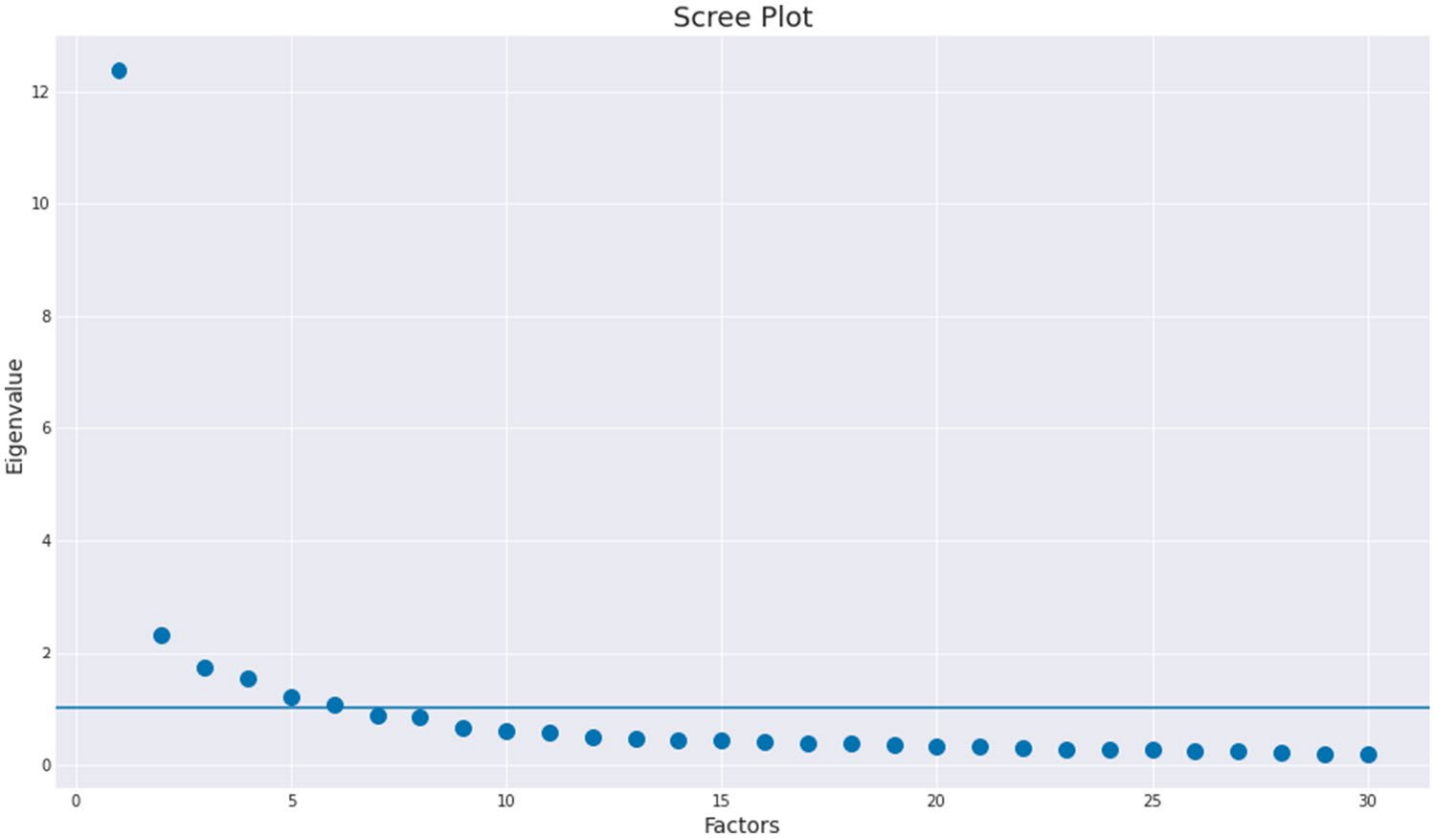
Scree plot of factor eigenvalues for a factor analysis of the co-responsibility items with no rotation. The choice of 6 factors satisfies both Kaiser criterion (the solid horizontal line is located at y = 1) and Cattell criterion (the so-called elbow method).

The selection of the number of factors was informed by the scree plot in Fig. 2, where the eigenvalues of a factor analysis without rotation are displayed. The choice of 6 factors satisfies both Kaiser and Cattell criteria: the 6th factor is located around the *elbow* of the curve and from the 7th factor onwards, the eigenvalues are smaller than 1.

Table 1 shows the summary of the subsequent factor analysis with 6 factors and *PROMAX* rotation. Factor names were selected based on insights from prior research on co-responsibility among bariatric surgery patients and their partners^14,15^ and in close collaboration with clinical partners. The loadings of the items on each of the 6 factors are shown in Table 2; the mapping between codes (I01, I02, …, I30) and items is also contained in the same table. The following constructs are identified:

1. Empowerment and support: I03, I04, I11, I15, I16, I18, I19, I20, I21, I22, I23, I24.
2. Relational aspects (Trust and security): I06, I07, I08, I09, I10.
3. Lack of sympathy: I17, I25, I26, I27.
4. Co-participation: I05, I28, I29.
5. Accepting help: I12, I13, I14.
6. Awareness: I01, I02.

**Table 1:** Summary of the exploratory factor analysis results (6 factors with PROMAX rotation).

	Factor 1	Factor 2	Factor 3	Factor 4	Factor 5	Factor 6
Sum of squares of loadings	6.82	2.63	2.23	1.97	1.84	1.16
Proportion of variance	0.23	0.09	0.07	0.07	0.06	0.04
Cumulative variance	0.23	0.31	0.39	0.45	0.52	0.55

The row labelled sum of squares of loadings shows the sum of squares of the loadings for each factor, which is then translated by into the proportion of variance explained (row named proportion of variance), and into the cumulative proportion of variance explained (row named cumulative variance).

**Table 2:** Factor loadings for each item of the co-responsibility sub-questionnaire.

	Factor 1	Factor 2	Factor 3	Factor 4	Factor 5	Factor 6
I01: My significant other’s activities and lifestyle choices affect mine (Awareness)	− 0.21	0.01	0.04	0.03	0.22	**0.63**
I02: My significant other is aware that his/her activities and lifestyle choices affect mine (Awareness)	0.24	− 0.01	0.02	− 0.09	0.08	**0.71**
I03: My significant other takes my lifestyle goals sufficiently into account in his/her behaviour (Empowerment and support)	**0.68**	0.20	− 0.07	− 0.04	− 0.23	0.21
I04: My significant other takes sufficient responsibility by actively helping me (Empowerment and support)	**0.73**	0.17	− 0.05	− 0.07	− 0.19	0.17
I05: I feel that my significant other and I are the same way in life (Co-participation)	0.16	0.37	− 0.07	**0.41**	− 0.11	0.10
I06: I feel sufficiently understood by my significant other regarding my lifestyle goals (Relational aspects)	0.35	**0.51**	− 0.01	0.04	− 0.16	0.09
I07: I feel accepted by my significant other for who I am, regardless of my appearance or weight (Relational aspects)	− 0.13	**0.85**	0.05	− 0.09	− 0.01	0.10
I08: I feel that my significant other cares about me as a person, whether I meet my lifestyle goals or not (Relational aspects)	− 0.17	**0.77**	0.07	− 0.02	0.20	− 0.09
I09: I think I can talk to my significant other about my feelings (Relational aspects)	0.14	**0.49**	0.05	0.04	0.26	− 0.08
I10: I feel I can talk openly with my significant other about my weight loss journey (Relational aspects)	0.13	**0.54**	0.04	0.03	0.26	− 0.11
I11: I feel support from my significant other in the decision to lose weight (Empowerment and support)	**0.59**	0.30	− 0.07	0.03	0.10	− 0.13
I12: I am open to help and support from my significant other in achieving my lifestyle goals (Accepting help)	0.03	0.26	0.00	0.07	**0.62**	0.03
I13: I ask my significant other for help in achieving my lifestyle goals when needed (Accepting help)	0.24	− 0.06	− 0.04	0.05	**0.63**	0.20
I14: I allow my significant other to help me (Accepting help)	0.13	− 0.00	0.01	0.03	**0.69**	0.17
I15: I experience sufficient support from my significant other with practical things that help me achieve my lifestyle goals (Empowerment and support)	**0.75**	0.09	0.00	− 0.01	0.08	− 0.09
I16: I find that my significant other listens enough when I talk about my lifestyle goals (Empowerment and support)	**0.55**	0.15	0.06	− 0.00	0.19	− 0.06
I17: I don't like the way my significant other addresses me about unhealthy behavior (Lack of sympathy)	− 0.11	0.04	**0.49**	− 0.04	0.12	0.08
I18: I find that my significant other makes enough time to assist me in achieving my lifestyle goals (Empowerment and support)	**0.80**	− 0.14	0.03	0.01	0.12	− 0.01
I19: I feel that my significant other has amassed enough knowledge to support me in achieving my lifestyle goals (Empowerment and support)	**0.64**	− 0.16	0.01	0.17	0.11	0.03
I20: I notice that my significant other reminds me not to eat unhealthy products (Empowerment and support)	**0.76**	− 0.18	− 0.20	0.01	0.06	0.01
I21: I feel encouraged enough by my significant other to achieve my lifestyle goals (Empowerment and support)	**0.98**	− 0.09	− 0.02	− 0.09	0.04	− 0.07
I22: I notice that my significant other is confident in my ability to achieve my lifestyle goals (Empowerment and support)	**0.68**	0.04	0.09	− 0.04	0.15	− 0.09
I23: I find that my significant other helps me enough to grow my confidence in my own abilities (Empowerment and support)	**0.69**	− 0.04	0.11	− 0.06	0.16	− 0.02
I24: I think I get enough compliments from my significant other for healthy behavior (Empowerment and support)	**0.68**	− 0.03	0.09	− 0.03	0.07	− 0.08
I25: I experience insufficient understanding of my significant other for the behavioral change needed to achieve my lifestyle goals (Lack of sympathy)	0.39	− 0.04	**0.53**	− 0.01	− 0.07	0.03
I26: I find that my significant other comments negatively on my behavior (Lack of sympathy)	− 0.02	0.03	**0.89**	− 0.07	0.02	− 0.02
I27: I find that my significant other complains that I am investing too much time and/or effort in achieving my lifestyle goals (Lack of sympathy)	− 0.07	0.10	**0.81**	− 0.01	− 0.08	− 0.03
I28: My significant other and I have the same lifestyle goals (Co-participation)	− 0.10	0.02	− 0.06	**0.99**	0.08	− 0.07
I29: My significant other and I work together to achieve the same lifestyle goals (Co-participation)	0.07	− 0.12	− 0.06	**0.80**	0.17	0.02
I30: I feel like my significant other isn’t trying to follow the same healthy behavior as me	0.16	− 0.10	0.37	0.30	− 0.19	− 0.02

The highest load for each item is highlighted in boldface. The first column shows the mapping between item code, item description, and identified construct. The last item did not load (in absolute value) on any factor by more than the 0.4 threshold. Hence, it is not assigned to any construct.

Item I30 did not load above the 0.4 threshold and was therefore not included in any construct and discarded from the questionnaire. The cumulative variance of all remaining 29-items across 6 factors is 55%.

Cronbach’s $\alpha$ revealed commonly a good internal consistency of the identified factors. ‘Empowerment and support' reports the highest value (0.933; 95% CI 0.927–0.939), while the lowest is reported by ‘Awareness' (0.664; 95% CI 0.620–0.704). Table 3 reports the value of Cronbach’s $\alpha$ for all the constructs identified above. High internal consistency was found also for the Close Persons Questionnaire (0.727; 95% CI 0.701–0.752). Notably, the 29-item scale for co-responsibility reports the highest value of $\alpha$ (0.943; 95% CI 0.938–0.948), see Table 4.

**Table 3:** Cronbach's $\alpha$ for the 6 constructs identified in the exploratory factor analysis.

	$\alpha$	95% CI
Factor 1: Empowerment and support	0.933	0.927–0.939
Factor 2: Relational aspects (Trust and security)	0.855	0.840–0.868
Factor 3: Lack of sympathy	0.794	0.773–0.814
Factor 4: Co-participation	0.829	0.809–0.846
Factor 5: Accepting help	0.841	0.823–0.857
Factor 6: Awareness	0.664	0.620–0.704

**Table 4:** Cronbach’s $\alpha$ for the following constructs: co-responsibility and spousal support.

	$\alpha$	95% CI
Co-responsibility	0.943	0.938–0.948
Spousal support	0.727	0.701–0.752

**Figure 3:**
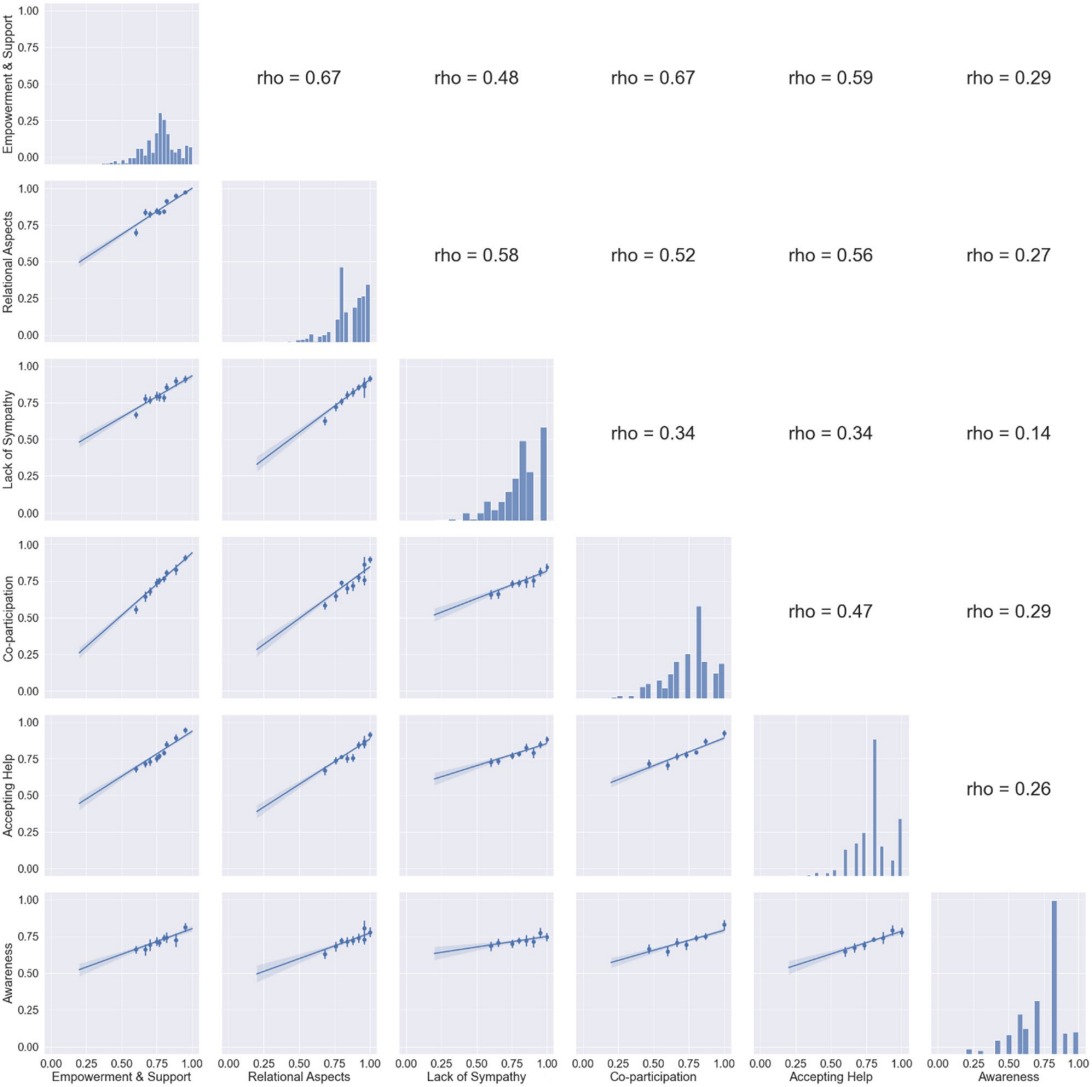
Pairwise relationships between the identified constructs. The plots on the diagonal show the histogram of the total score; in the lower triangle are pairwise binned scatterplots with linear fit; in the upper triangle, pairwise Pearson’s correlation coefficients. The score of each construct is normalized between 0 and 1 to allow easier comparison. Binned scatterplots are drawn by dividing datapoints on the x-coordinate in 10 groups of equal size and computing the mean of the y-coordinate. Pearson’s $\rho$ coefficients correspond to the plot that is symmetric with respect to the main diagonal, and they are directly linked to the R^2^ coefficient of the corresponding linear fit.

The pairwise relationships between the identified constructs are shown in Fig. 3. The binned scatterplots show that an increase in the score of a construct commonly correspond to an increase in the score of other constructs, too. ‘Empowerment and support' and ‘Relational aspects' show a linear fit (with each other and) with ‘Lack of sympathy', ‘Co-participation', and ‘Accepting help' that is inclined at about 45 degrees. This suggests a mutual increase of about the same magnitude for those constructs. However, the pairwise Pearson’s correlations are nearly always below 0.6, indicating that (i) the relationship between factors might not be best approximated by a linear function and (ii) the fit is degraded for low scores. Finally, ‘Awareness' shows low values of correlations (< 0.3) with any other constructs.

It can be noted that the correlation between ‘Lack of sympathy' and other constructs are positive, even though the meaning of the former construct is negative in nature. This is a natural consequence of the inversion to the answers from negatively phrased items, which compose the construct.

## Discussion

In this paper we present a valid tool to measure co-responsibility, report psychometric properties and the underlying factor structure.

The 29-item co-responsibility (CoReCare) questionnaire shows high internal consistency and Pearson correlations indicate good construct validity with spousal support^16^, which was selected as a construct that was most closely narrated. Furthermore, we have identified six factors that explain a large amount of variance in co-responsibility (55%), and show high internal consistency: ‘Empowerment and support', ‘Relational aspects (Trust and security)', ‘Lack of sympathy', ‘Co-participation', ‘Accepting help', and ‘Awareness'. Moreover, all factors are positively associated to each other as shown by pairwise correlations. However, pairwise correlations are predominantly below 0.6, suggesting an inherently noisy relationship and indicating a potentially weak predictive value from one construct to the other. ‘Awareness' showed the lowest correlation (< 0.3) with all other constructs. Being a small factor (2 items) with low association to the other constructs, ‘Awareness' does not substantially influence the co-responsibility score. Thus, while ‘Awareness' can be seen a required first step towards acting co-responsible, it does not guarantee patient-partner co-responsibility.

## Limitations and future research perspectives

Three main limitations need mentioning. First, although the questionnaire is meticulously composed, no test-retest reliability could be established due to the sample being anonymized. Second, participants were randomly, yet carefully sampled in the general population according to pre-defined inclusion criteria. While all participants indicated to have either a partner, friend or family member who supported their weight loss journey, we had no sight on the intensity and nature of the support. Since the intent was to measure ‘perceived’ co-responsibility, the nature or actual intensity might be less relevant but could have added unexplained variance to the results. Third, while we targeted individuals who tried to lose weight, we did not specify a minimum requirement for weight loss. Therefore, this questionnaire should be validated among the clinical target population who likely have more ambitious weight loss goals^28^.

We propose four main avenues for future studies. First, co-responsibility can be used to understand social dynamics and help to identify areas to improve the experience of remote care settings. The need and value of assessing PREMS, for example in bariatric care, has been recently evidenced^29^. However, to draw conclusions on patient populations, the questionnaire needs to be validated among a clinical sample. Second, to enclose all three elements of the triangle as proposed by Neutelings et al.^11^, future research should investigate how patients perceive co-responsibility of health care professionals. Third, significant others should be included to understand how co-responsible they behave. Fourth and last, understanding patient experience and factors contributing to it, is an objective and proof-point of VBC. Co-responsibility would gain more relevance when a link with improved health outcomes could be established. If higher co-responsibility were indeed found to be associated with better health outcomes, significant others, or other primary care givers, could turn out to be a strong asset in facilitating a patients care journey and in achieving their health goals.

In summary, the CoReCare questionnaire is clinically relevant as it could be positioned as a valuable tool that goes beyond traditional patient-reported outcome measures (PREMS/PROMS) by considering the broader context of patient relationships and providing actionable information for healthcare professionals to enhance the quality of care in out-of-hospital settings.

## Conclusion

We propose co-responsibility as a new construct to understand and potentially improve social dynamics to achieve desired health outcomes in remote care settings. Therefore, we present a 29-item CoReCare questionnaire which reflects co-responsibility across 6 factors: Empowerment and support, Relational aspects (Trust and security), Lack of sympathy, Co-participation, Accepting help, and Awareness. This questionnaire can be used as an extension of current PREMS when aiming to assess and improve the patient experience of a care episode outside of the hospital. Finally, insights from the questionnaire can inform the development of a program for remote care focused on long-term weight maintenance.

## Data Availability

The datasets used and/or analyzed during the current study available from the corresponding author on reasonable request.
